# Characterization of Marigold Flower (*Tagetes erecta*) Extracts and Microcapsules: Ultrasound-Assisted Extraction and Subsequent Microencapsulation by Spray Drying

**DOI:** 10.3390/foods13152436

**Published:** 2024-08-01

**Authors:** Nilar Oo, Khursheed Ahmad Shiekh, Saeid Jafari, Isaya Kijpatanasilp, Kitipong Assatarakul

**Affiliations:** 1 Department of Food Technology, Faculty of Science, Chulalongkorn University, Bangkok 10330, Thailand; nilaroo2018@gmail.com (N.O.); khursheedahmad.s@chula.ac.th (K.A.S.); saeid.j@chula.ac.th (S.J.); ykijpat@gmail.com (I.K.); 2 Department of Food Science, College of Agriculture and Life Sciences, Cornell University, Ithaca, NY 14850, USA

**Keywords:** response surface methodology, ultrasonication, marigold flower, polyphenols, microcapsules, antimicrobial activity

## Abstract

Ultrasound-assisted extraction using response surface methodology was employed to extract marigold flower, resulting in a marigold flower extract (MFE) with elevated levels of total phenolic compounds (TPCs), total flavonoid content (TFC), total carotenoid content (TCC), and antioxidant activity, as assessed by 2,2-diphenyl-1-picrylhydrazyl (DPPH) and ferric reducing antioxidant power (FRAP) assays, under conditions of 40 °C temperature, 15 min extraction time, and 68% ethanol concentration. The MFE was subsequently encapsulated using spray drying with 45% maltodextrin (MD) (MFE–MD; 1:1, 1:2) and 20% gum arabic (GA) (MFE–GA; 1:2, 1:3). The MD (1:2 ratio) sample showed the highest encapsulation yield, while the 45% MD (1:1 ratio) sample exhibited the highest encapsulation efficiency (*p* ≤ 0.05). Samples containing 45% MD (1:1 ratio) and 20% GA (1:2 ratio) had the highest moisture content, with the 45% MD (1:1 ratio) sample showing the lowest water activity (*p* > 0.05). These samples also displayed higher L* and a* values compared to the 20% GA samples, which had increased b* values (*p* ≤ 0.05). Micrographs of the 20% GA (1:3 ratio) and 45% MD (1:2 ratio) samples revealed spherical shapes with smooth surfaces. The 20% GA (1:2 ratio) microcapsules exhibited the highest total phenolic content (TPC) among the samples (*p* ≤ 0.05). Thus, ultrasound-enhanced extraction combined with response surface methodology proved effective in producing functional food ingredients from plants.

## 1. Introduction

Consumers’ demand towards functional food ingredients that could enhance the therapeutic properties of food products is increasing worldwide [1]. Functional food ingredients could serve as a reliable substitute to chemical additives in food and pharmaceutical industries to prevent non-communicable diseases [2]. Phytochemicals attained from plant sources such as stem, leaf, flower, and fruit waste, including seed, have been investigated and shown to exhibit enormous bioactive properties [3]. The total phenolic profiles of plant-based extracts have been reported, with higher antioxidant and antimicrobial properties [4]. For the extraction of bioactive compounds, aqueous ethanol as a safe solvent has been employed in conjunction with non-thermal technologies. Moreover, novel extraction technologies might minimize the loss of active ingredients during extraction, preparation, and fortification processes for functional food development [5].

Flower petals are a rich source of bio-colorants and a diversity of polyphenols. Marigold (*Tagetes* spp.) is a species of the Asteraceae family abundant in carotenoids such as lutein, fatty acid esters, and diesters [6]. Extracts from marigold flower have been used for culinary and medicinal purposes since the Middle Ages. Marigold flowers and leaves contain physiologically active phytochemical essential oils that are employed in a variety of illnesses viz skin allergies, wounds, burns, kidney problems, and menstrual irregularities [7]. Marigold petals are yellow-to-orange colored due to the presence of flavonoid, essential oil, and other pigments employed in the Ayurveda system of medicine against several physiological and neurological disorders [8]. Additionally, Tagetes species are yellowish and used as bio-colorants in culinary services [9].

Plant-based biological materials can be extracted using conventional (Soxhlet, maceration, and hydro-distillation) and novel methods (ultrasonication, enzyme, and microwave, pulsed electric field, supercritical fluid, and pressurized liquid extraction technologies) [10]. Ultrasound-assisted extraction (UAE) could minimize the processing time and boost the extraction rate and production in comparison with conventional methods [11]. UAE with frequencies between 20 kHz and 100 MHz has physical specifications such as ultrasonic power, temperature, and processing time, with an impact not only on the extraction yield but also on the extract composition [12]. Response surface methodology (RSM) generally utilizes a second-order polynomial model commonly used to describe the interaction between the response variables and the independent variables. The second-order model includes both linear and quadratic terms of the independent variables, such as extraction time, temperature, and solvent concentration, as well as interaction terms [13].

Microencapsulation is a valuable technique for the food and beverage industries, helping protect sensitive ingredients such as vitamins, minerals, flavors, and active compounds from degradation caused by exposure to light, oxygen, moisture, and other environmental factors [14]. It can also enhance the stability, shelf life, and bioavailability of these ingredients. Among the various methods of microencapsulation, spray drying is a widely used technology because of its affordability, scalability, and versatility. Spray drying involves atomizing a liquid mixture containing the core material and the coating material into droplets, which are then dried by hot air to form small, dry, free-flowing particles [15].

Several studies have been published on spray-dried microcapsules containing plant extracts. However, from the current status of the scientific literature, there are no records available on RSM-optimized ultrasonic extraction as well as encapsulating the optimized extract of marigold flower petals. Therefore, marigold flower extract was prepared and characterized by its physicochemical properties without and with encapsulation in different ratios of maltodextrin and gum arabic. Moreover, the bioactivity of MFE was measured, and microstructural analysis was performed for microcapsules. In general, this study focused on the development of functional MFE microcapsules for future food applications.

## 2. Materials and Methods

### 2.1. Sample Procurement and Preparation of Marigold Flower Powder

Marigold flower samples from a fresh flower market in Bangkok, Thailand, were bought in the month of August 2022, and were brought to Food Technology Laboratory, Chulalongkorn University. On arrival at the laboratory, marigold flower petals were gently rinsed with distilled water to remove dirt. The cleaned petals were placed in a hot air oven (Memmert, DO 6062, Memmert GmbH & Co. KG, Dchwabach, Germany) for 10 h at 60 °C to adjust the moisture content to <5%. The dried sample was blended and screened using a sieve (50-mesh size) to obtain marigold flower powder (MFP) and stored in aluminum-laminated bags at −20 °C for further analysis.

### 2.2. Response Surface Methodology, Optimization, and Preparation of Marigold Flower Extract

The RSM technique equipping Box–Behnken design (BBD) was applied for the maximum extraction condition on MFP samples using various independent variables, as shown in Table 1. The three independent variables were ethanol concentration (A), temperature (B), and time (C), which presented three levels (−1, 0, 1) in numeral form, as shown in Table 1. Briefly, MFP sample (3 g) was mixed with 100 mL (60%, 80%, and 100%) of ethanol and the beakers were placed in an ultrasonic bath (Elmasonic bath), followed by heating at various temperatures (30, 40, 50 °C) and process times of 5, 10, 15 min, respectively (Table 2). The ultrasound power was set at 70 W/mL. A refrigerated centrifuge (Centrifuge Kubota, series 6000, Osaka, Japan) was used for the separation of ethanolic extract from the solid residue of all the 17 samples from RSM analysis, set at 10,000 rpm for 10 min. Subsequently, a rotary evaporator (Oilbath B-485, BUCHI, Flawil, Switzerland) was used at 45 °C to obtain ethanol-free marigold flower extract (MFE). Then, 10 mL of distilled water was added to the residual MFE samples and held in brown-colored glass bottles at 4 °C for further analysis.

All 17 sample extracts prepared from RSM values of independent variables were subjected to analysis. The optimum extraction condition of MFE was characterized based on the highest bioactive compounds assayed by total phenolic compound, total flavonoid content, carotenoids content, and antioxidant properties detailed in Section 2.5.1.

### 2.3. Antimicrobial Activity of RSM-Optimized MFE

#### 2.3.1. Growth Conditions

Microbial cultures, including *Escherichia coli* (*E. coli* ATCC 25922) and *Staphylococcus aureus* (*S. aureus* ATCC 25923), were obtained from Faculty of Science, Chulalongkorn University, and inoculated in 10 mL of sterile Muller–Hinton Broth (MHB) to obtain inoculum for bacterial culture. The inoculated test tubes containing MHB were grown for 18–24 h at 37 °C. The initial load of the test microorganism was approximately 6.0 log CFU/mL by measuring the optical density at 600 nm previously described by Saénz et al. [16].

#### 2.3.2. Determination of Inhibition Zones of MFE by Minimum Inhibitory Concentration and Disk Diffusion Method

The antimicrobial activities of RSM-optimized MFE were determined using the disk diffusion technique [17]. To obtain the final concentration, a stock extract solution was made by dissolving it with relative solvents (ethanol). The sterile, blank disks were soaked with 20 µL of extract and then dried. These disks were placed on Mueller–Hinton agar plates that had previously contained 108 CFU/mL of the target bacteria. To test the inhibitory zone of the MFE samples, ethanol and chloramphenicol were utilized as a negative control and positive control in each disk, correspondingly. The plates containing the treated disks were incubated for 24 h at 37 °C. Zones of inhibition were determined as diameter and expressed in mm [18]. An antibiotic (chloramphenicol, 32 µg/mL) was used to compare the antimicrobial potential of the MFE.

The optimized MFE sample (5 mg/mL) was mixed with sterile distilled water. Two-fold dilutions of 5, 4, 3, 2, 1, 0.5, and 0.25 mg/mL from the previous solution were then made in sterilized water.

A bacterial inoculum was prepared at 37 °C for 16 h, and Mc Farland 0.5 standard was used to assess the turbidity of both the bacterial cultures. Additional serial dilutions in sterile MHB were carried out to achieve a suitable suspension with at least 1 × 10^−6^ CFU/mL. The inoculum (50 μL) of cell cultures was transported to a sterile 96-well plate with 100 μL of MHB. Extract dilutions in 100 μL were added. A positive control (containing only microbial inoculum) was poured into each micro-plate well. The micro-plates were incubated at 37 °C for 24 h. The OD value at 600 nm was measured with a microplate reader. The MIC value indicated no visible growth of the tested bacterial strain.

### 2.4. Microencapsulation of RSM-Optimized MFE in Maltodextrin (MD) and Gum Arabic (GA)

The RSM-optimized (68% ethanol concentration, 40 °C temperature, and 15 min) MFE sample was microencapsulated via spray drying in 45% (*w*/*v*) MD (1:1; and 1:2 ratios of MFE to MD), and 20% (*w*/*v*) GA (1:2; and 1:3 ratios of MFE to GA). Microcapsules were prepared by mixing the optimized MFE sample with the coating material at different ratios. The solution for spray drying was prepared on a magnetic stirrer for 5 min and homogenized. The nozzle size (0–5 mm) of the spray drying machine was employed in the microencapsulation of MFE in MD and GA used as wall materials. The MD and GA solutions containing MFE were fed into the spray dryer (inlet air temperature of 155 °C and outlet air temperature of 90 °C) to obtain MFE-encapsulated powder in MD and GA. The powders were collected and kept at −20 °C in aluminum-laminated bags until further analysis.

### 2.5. Physiochemical Properties of MD and GA Microcapsules Loaded with MFE

The encapsulation yield (%) of MFE microcapsules was determined as described by Ramakrishnan [19]. A water activity (aw) analyzer (model MS1, Novasina, Lachen, Switzerland) was used to determine the aw of the MFE microcapsules. A moisture analyzer determined the moisture content (%). The aluminum cups were dried at 105 °C for 2–3 h, put in a desiccator until the temperature of the container was equal to the room temperature, and then weighed. The procedure was repeated until the weight obtained was constant. For sample analysis, 0.2 g of a sample was put into the aluminum cup and dried at 105 °C for 4–5 h. Aluminum cups were transferred from the hot air oven to the desiccator, cooled down, and the final weight was measured after several repetitions. The moisture content was figured out based on Equation (1):

Moisture (%) = (B − A)/B × 100(1)

where ‘B and A’ are the sample weights before and after drying, respectively.

A CIE LAB system (L*, a*, and b*) using a chroma meter Minolta CR-400 color meter was employed at room temperature to assess the color values of the MFE microcapsules. A scanning electron microscope (SEM) and energy dispersive X-ray spectrometer were applied to examine the surface characteristics of the microcapsules (JEOL, JSM-IT300 Oxford, X-Max N 20) at 30 kV magnifications at 1000 times.

The encapsulation efficiency (%) was evaluated by Saénz [16]. Further, 0.1 g of MFE microcapsule powder was weighed and dissolved in 1 mL of a mixed solution (ethanol–acetic acid–water) at a ratio of (50:8:42) and vortexed for 1 min. The sample was centrifuged at 10,000 rpm for 5 min and then the residue was separated using Whatman No. 1. Surface bioactive compounds were calculated by using 0.1 g of MFE-encapsulated samples, which were weighed and dissolved in the mixed solution (ethanol and methanol at a ratio of 1:1) and vortexed for 1 min. The encapsulation efficiency of GA- and MD-based microcapsules was calculated by Equation (2):

Efficiency % = TO − SO/TO × 100 (2)

where TO is the total bioactive compounds, and SO is the surface bioactive compounds.

Water solubility was investigated as described by Sarabandi [20]. Briefly, sample powder (1 g) was put into distilled water (100 mL) and mixed at 400 rpm for 4 min using a magnetic stirrer. The samples were centrifuged at 4000× *g* for 4 min. Then, 25 mL of the supernatant was placed in a pre-weighed plate and placed in oven at 105 °C for 5 h. The solubility was obtained by dividing the weight of the dried supernatant by the initial powder weight.

#### 2.5.1. Determination of Bioactive Compounds (TPC and TFC), Antioxidant Activity (DPPH and FRAP), and Carotenoids of MFE without and with Encapsulation

MFE (0.5 mL) and MFE microcapsule (1 g) were added into 10 mL of distilled water. The MFE microcapsule was mixed with distilled water (10 mL) and vortexed for 3 min. The sample was placed on a shaker at 30 °C for 30 min. After centrifugation at 4000 rpm for 20 min, the MFE microcapsule supernatant was collected. The MFE solution and MFE supernatant solution were subjected to the analysis of TPC, TFC, carotenoid content, DPPH, and FRAP, detailed as follows.

The Folin–Ciocalteau method as presented by Jafari et al. [17] was used to determine the TPCs of the MFE samples without and with microencapsulation. Further, 0.5 mL of each MFE and supernatant of MFE microcapsule were diluted in 10 mL of distilled water in an amber vial and mixed with 10% Folin–Ciocalteau’s phenol reagent (0.5 mL). After the incubation time of 5 min was over, 10% (*v*/*v*) sodium carbonate (2 mL) was poured into the MFE samples, and they were kept in a dark room for 10 min. Finally, readings of all the samples were taken at 765 nm using a spectrophotometry method and values were calculated as mg GAE/100 g db using gallic acid as the standard.

The TFC was investigated according to aluminum tri-chloride, as described by Jafari [17]. In detail, 1 mL of MFE and supernatant of the MFE microcapsule samples were pipetted and added to 1 mL of 2% AlCl_3_ and subjected to incubation for 10 min in a dark room. The absorbance of the samples was recorded at 430 nm in a spectrophotometer and calculated as mg QE/100 g db using quercetin as the standard.

The carotenoid content of MFE and MFE microcapsules was analyzed according to Biswas et al. [21], with some modifications. In detail, 250 µL of MFE and supernatant of the MFE microcapsule samples were pipetted and mixed with 5 mL of acetone, and the sample mixtures were centrifuged at 1370× *g* for 10 min. After that, the supernatant was collected and the remaining samples were subjected to the extraction again using acetone (5 mL). Finally, both supernatants were collected after filtration. Readings of all the samples were noted at 450 nm, with a spectrophotometry method using acetone as a blank, and revealed as mg carotenoid/100 g db.

The antioxidant activity of the MFE and MFE microcapsules was investigated according to the DPPH method, as described by Brand-Williams et al. [22]. In detail, 250 µL of MFE and supernatant of MFE microcapsule samples were pipetted and blended with 4.75 mL of DPPH, and the mixture solution was kept in a dark room for 15 min for incubation. The absorbance of the samples (A_final_) was measured at 515 nm in a spectrophotometer using distilled water as a blank. The difference in the absorbance values (A_diff_.) was indicated as mM Trolox/100 g dry wt; (Equation (3)):

A_diff._ = A_initial_ − A_final_ (3)

The antioxidant activity of MFE and MFE microcapsules was calculated by the FRAP method as described by Benzie and Strain [23]. In detail, 50 µL of MFE and supernatant of the MFE microcapsule samples were pipetted and mixed with 950 µL of FRAP for 4 min in a dark room. The absorbance of the samples (A_final_) was measured at 593 nm in a spectrophotometer using distilled water as a blank. The difference in the absorbance values (A_diff_.) were calculated as mM Trolox/100 g dry wt; (Equation (4)).

A_diff._ = A_final_ − A_initial_ (4)

### 2.6. Statistical Analysis

Using the Design Expert 11 program, the Box–Behnken design was implemented to approximate the optimum condition from the UAE experiment (Stat-Ease, Inc., USA). Three-dimensional (3D) model designs were also created using Design Expert 11 software. All physical and chemical analyses were conducted in triplicates (*n* = 3), and data were interpreted with SPSS version 20.0 statistic software. Duncan’s multiple range test was applied to demonstrate significant differences (*p* ≤ 0.05) among the microencapsulated samples in a one-way variance analysis (one-way ANOVA).

## 3. Results and Discussion

### 3.1. Optimization of Ultrasound-Assisted Extraction (UAE) of Bioactive Compounds from Marigold Flower Using RSM

Plant-based products including fruits, vegetables, and their byproducts that are discarded as waste contain abundant amounts of polyphenols, showing antioxidant and antimicrobial activities. The antioxidant potential of plant-based phenolic compounds aids in the reduction of free radicals via hydrogen donation mechanisms to prevent the malfunctioning of life processes in the human body [24]. After phytochemical characterization, the MFE sample extracted from the optimized condition at 68% ethanol concentration, 40 °C temperature, and 15 min of extraction time had the highest phytochemical content and antioxidant properties, and it was subjected to antimicrobial analysis prior to the microencapsulation process. As indicated in Table 2, the highest TPC value in the marigold flower extract (MFE) was attained in the 12th run sample set at 5 min, 40 °C, and 100% ethanol concentration, compared to the 16th sample set at 10 min, 30 °C, and 60% ethanol concentration. Three independent variables including time, temperature, and ethanol concentration showed a linear effect on the extraction of TPCs from MFE, with marked significance (*p* ≤ 0.05). Table 3 presents the ANOVA results of significant differences in linear, quadratic, and their interaction terms as indicated by A, B, and C on the response values (Y). The model’s fit values indicate the genuine form of the computed response surface plot. However, a lack of fit was insignificant in all the three models (Table 3). The R^2^ values in the range of 0.60–0.97 reveal that all the three models were best-suited to the response. The experimental results obtained in TPCs following the predicted models were in agreement with the results of Yıkmış [25]. Table 4 displays the regression coefficients of the predicted second-order polynomial models for TPC, TFC, carotenoid content, and antioxidant activity by the DPPH and FRAP assays using BBD. The second-order polynomial model’s equilibrium, suggesting the findings of MFE samples with varied conditions of time, temperature, and ethanol concentration, is given as follows:

TPC = −339.07602 − 0.423366 A + 19.64846 B − 0.740277 C + 0.003297 A × B − 0.033654 A × C + 0.001099 B × C + 0.004670 A^2^ − 0.224864 B^2^ + 0.129665 C^2^

**Figure 1:**
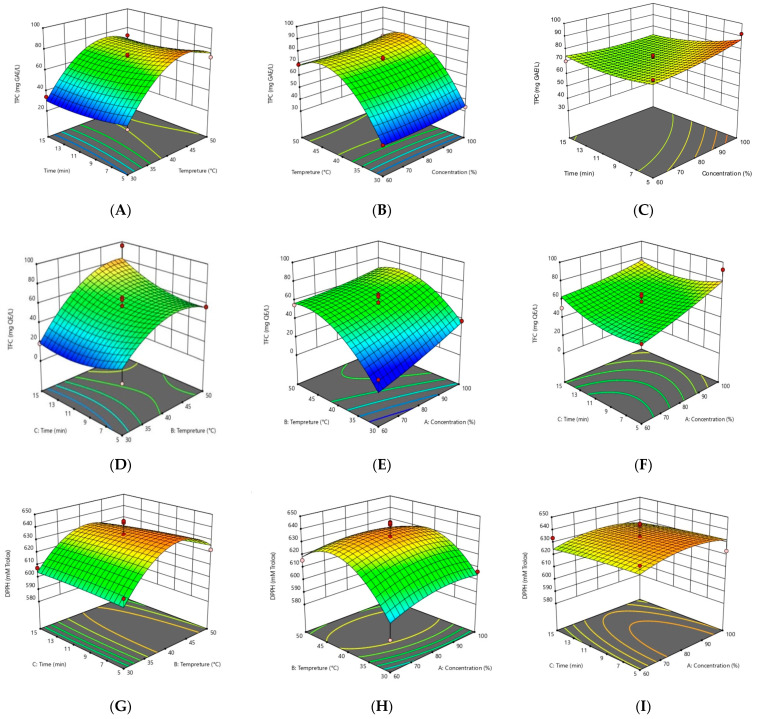

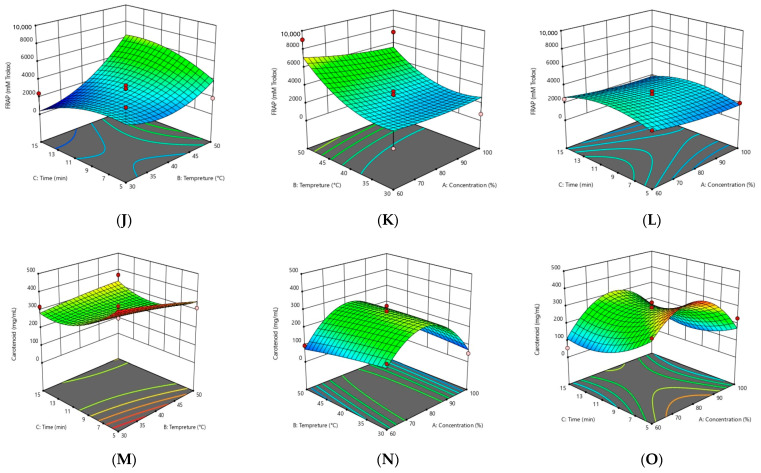
Three-dimensional plots of response surface methodology of total phenolic compounds, total flavonoid compounds, antioxidant activity by 2,2-diphenyl-1-picrylhydrazyl (DPPH) and ferric reducing antioxidant power (FRAP) assays, and carotenoid content as a function of significant interaction between factors; (**A**,**D**,**G**,**J**,**M**) temperature and time; (**B**,**E**,**H**,**K**,**N**) temperature and ethanol concentration; (**C**,**F**,**I**,**L**,**O**) time and ethanol concentration of MFE. The red dot represents design point above predicted value. The pink dot represents design point below predicted value.

Figure 1A–C demonstrates 3D graphic surface plots for the TPCs obtained during the RSM optimization of MFE samples using A, B, and C independent variables (Table 1). In this Figure 1A, the temperature and time combination yielded the lowest TPC values at 30 °C. The higher increments in TPC values were attained with the increase in UAE processing time and temperature. Kobus et al. reported that temperature has been shown to alter extraction efficiency by changing diffusion efficiency and solvent solubility [26]. Therefore, temperature increases the diffusion coefficient, which accelerates the diffusion rate and TPC values. Figure 1B shows the results of TPCs of MFE as a function of temperature and ethanol content. The experimental results from the 3D graphics demonstrated that increasing the ethanol content from 60% to 100% (*v*/*v*) decreased the TPC values. These findings could be clarified by the ease with which water and low concentrations of ethanol (i.e., 60% *v*/*v*) may penetrate cells to dissolve phenolic compounds, in contrast to high concentrations of ethanol, which were frequently discovered to decrease extraction rates, leading to protein denaturation, which prevents the dissolution of phenolic compounds [27]. Furthermore, the effects of ethanol concentration and time on the TPC values of the MFE samples are revealed in Figure 1C, which indicates that increasing the processing time tended to increase the extraction efficiency of TPCs. The UAE process is an efficient technology for the extraction of high-quality intracellular compounds in a short period of time, as stated in Wen et al. [25].

The findings from Table 2 reveal that the minimum TFC values were evaluated at the condition (10 min, 30 °C, and 60% ethanol), while the maximum TFC value was obtained at a condition of 80% ethanol (*v*/*v*), 50 °C, and 15 min. Temperature, time, and ethanol concentration showed statistically significant linear impacts on the TFC values in the MFE samples (*p* ≤ 0.05) (Table 3). The linear effect of ethanol and temperature and the quadratic effect of temperature had significant effects on the TFC values. The equilibrium of the second-order polynomial model, expressing the effect of temperature, time, and ethanol concentration on the TFC value using RSM analysis, was characterized as follows according to the following equation:

TFC = −361.50901 + 1.44252 A + 16.94897 B − 9.66077 C − 0.028197 A × B − 0.046088 A × C + 0.190649 B × C + 0.004534 A^2^ − 0.180147 B^2^ + 0.319107 C^2^

The TFC lack of fit test was insignificant (*p* > 0.05), which can be expressed as the proposed regression equation generating less errors when comparing findings from the experiments, and the independent variables had substantial effects on the outcomes. The coefficient of determination (R^2^ = 84.35), as well as the adjusted coefficient of determination (R^2^ Adj = 64.22) (Table 3), showed a high degree of fit and appropriateness in predicting experimental results.

In Figure 1D, the effects of time and temperature on the amount of TFC in the MFE samples indicated that the TFC values of the MFE sample were minimum at 30 °C. The TFC values increased with increasing extraction time because a longer extraction time can increase the mass transfer velocity and then release the bioactive compounds from the plant matrix by destroying the plant cells. Figure 1E shows how the TFC values were slowly increased with increasing ethanol concentration until 80% *v*/*v*. Cisowska et al. also confirmed that in comparison to only water or pure ethanol, ethanol solutions with some water, especially those with a concentration of 40–80% ethanol, were more effective at extracting polyphenolic compounds [26]. Figure 1F shows that the lowest extraction time for the TFC value was 10 min; then, it increased continuously, and the maximum value was 15 min. Ethanol is a polar solvent that is used to extract bioactive chemicals from plant matter. Factors that affect the extraction efficiency of phenolic compounds also have an impact on the extraction of flavonoid compounds, according to Salehi et al. [28]. The second-order polynomial model, describing the effect of temperature, time, and ethanol content in equilibrium on antioxidant activity by the DPPH assay of MFE using RSM analysis, is described as follows:

DPPH = +84.32812 + 3.04102 A + 19.81250 B + 2.60156 C − 0.017578 A × B − 0.003125 A × C − 0.051563 B × C − 0.013867 A^2^ − 0.212500 B^2^ − 0.034375 C^2^

Table 2 shows that the lowest antioxidant activity using the DPPH assay was found at 60% ethanol concentration (*v*/*v*), 30 °C, and 10 min, while the highest amount was revealed at 40 °C and 80% ethanol concentration (*v*/*v*) for 10 min. According to the ANOVA, the quadratic effect of temperature utilized in the MFE samples in the DPPH assay values was statistically significant (*p* ≤ 0.05). Meanwhile, the linear effects of temperature, time, and ethanol concentration and the relation between temperature and time, time and ethanol concentration, temperature and ethanol concentration, and the quadratic effect of concentration and time on the antioxidant activity of the MFE samples applied by using the DPPH assay was statistically insignificant (*p* > 0.05). Because the lack of fit test for DPPH values was insignificant (*p* > 0.05), the model was well fitted. The coefficient of determination (R^2^ = 73.46) and adjusted coefficient of determination (R^2^ Adj = 39.34) demonstrate a high degree of fit and appropriateness in predicting experimental results (Table 3). The high R^2^ values indicate that the quadratic model was very effective at fitting the data, and the adjusted R^2^ (R^2^ Adj) indicates that the predicted and experimental results of the model are in good agreement.

In addition, the surface plots of the three-dimensional responses of the antioxidant activity of MFE samples by the DPPH assay are demonstrated in Figure 1G. Figure 1G indicates the effects of extraction time and temperature on antioxidant activity using the DPPH assay, and the antioxidant activity increased with increasing time. Figure 1H depicts the effects of ethanol concentration as well as temperature on DPPH. The antioxidant activity of DPPH improved when the temperature was raised to 40 °C, but declined when the temperature was raised to 50 °C. When the ethanol concentration was raised by more than 80% (*v*/*v*) in Figure 1I, the antioxidant activity in the DPPH assay began to decline. This research is also in line with Liyana-Pathirana and Shahidi [29], who discovered that utilizing ethanol concentrations of 60 to 80% (*v*/*v*) resulted in better antioxidant activity than using the same ethanol concentration with a longer extraction period, as evidenced by the antioxidant activity in the DPPH assay. It is also accepted that the extraction period must be long enough to avoid bioactive components degrading and resulting in reduced antioxidant activity [30].

The linear impact of temperature on the antioxidant activity in MFE samples by the FRAP assay was statistically significant (*p* ≤ 0.05). On the other hand, the quadratic effects and relationship of time, temperature, and ethanol concentration on FRAP values in the MFE samples were statistically insignificant (*p* > 0.05) (Table 3). The equilibrium of the second-order polynomial equation describing the effect of temperature, time, and ethanol concentration on the antioxidant activity of MFE samples as measured by FRAP values was as follows:

FRAP = +24,493.26316 − 56.58947 A − 1025.17895 B − 282.90526 C − 1.74474 A × B + 0.447368 A × C + 24.60421 B × C + 0.622763 A^2^ + 13.68895 B^2^ − 37.01684 C^2^

The quadratic model’s high R^2^ values indicate that the data fitted well under experimental conditions, and the modified coefficient of determination reveals the model’s adequacy in predicting experimental findings as well as its high degree of fit. The minimum antioxidant activity measured by the FRAP value was discovered at 100% ethanol concentration at 30 °C for 10 min, whereas the greatest FRAP value was obtained at 50 °C and 60% ethanol concentration for 10 min. Surface plots of three-dimensional responses for the effects of temperature and time, temperature and ethanol concentration, and ethanol concentration and time are shown in Figure 1. The results from Figure 1J demonstrate that antioxidant activity did not show any significant changes when the temperature rose from 30 °C to 40 °C. However, when the temperature rose to over 40 °C, the antioxidant activity by the FRAP assay increased. Consistently, it was discovered that as the temperature rose from 20 °C to 50 °C, the extraction rate increased and then fell as the temperature rose even higher [31]. High temperatures accelerate mass transfer, enhance solubility, and reduce surface tension and viscosity. Nevertheless, extreme heat can cause the decomposition of phenolic compounds, which reduces antioxidant activity [32]. Figure 1K demonstrates that increasing the ethanol concentration caused a decline in antioxidant activity by FRAP values. Additionally, Figure 1L indicates the effect between ethanol concentration and time and shows that the minimum value of antioxidant activity was calculated at 10 min with 100% ethanol concentration (*v*/*v*).

According to the analysis, the linear effects of time and the quadratic effects of ethanol concentration and time applied to the MFE samples on carotenoid were statistically significant (*p* ≤ 0.05) (Table 3). The remaining quadratic effect and the interaction between temperature, time, and ethanol concentration shown on carotenoid content in the MFE samples were not statistically significant (*p* > 0.05) (Table 3). The equilibrium of the second-order polynomial model, describing the effect of temperature, time, and ethanol concentration on the total carotenoid content (TCC) of MFE using RSM analysis, was described as follows:

TCC = −1115.00719 + 61.66381 A − 16.48538 B − 125.44919 C + 0.149270 A B + 0.360736 A × C + 0.753262 B × C − 0.450316 A^2^ − 0.054940 B^2^ + 2.85963 C^2^

The lowest TCC was discovered at 100% ethanol concentration and 30 °C for 10 min, while the greatest TCC value was recorded for 5 min at 30 °C and 80% ethanol concentration. Surface plots of the three-dimensional responses for the effects of temperature and time, temperature and ethanol concentration, and ethanol concentration and time are shown in Figure 1. The effect of extraction time and temperature on TCC is seen in Figure 1M, where the TCC value increased as the extraction period increased because long extraction periods produce more cell wall breaking due to ultrasound. The duration of the extraction process is crucial in the extraction of carotenoids as prolonging the processing time of the solvent with the solids may promote the diffusion of the compounds and result in carotenoids being more easily discharged from the matrix into the extraction medium. Similar to that, the ethanol concentration is crucial to achieving the most carotenoids back. Figure 1N demonstrates that increasing the ethanol concentration up to 80% resulted in increased carotenoid content, which then decreased when the ethanol concentration increased to 100%. This is possibly because ultrasound waves spread more widely in aqueous solutions, and using a solvent with water can lead to greater radical production because of the ultrasound-induced dissociation of water. The extraction efficiency of the target chemicals may reduce because the oxidative reaction and the extraction reaction can coexist [33]. In Figure 1O, it can be seen that the lowest extraction time for the TCC value was 10 min; then, it gradually increased until 15 min.

The second-order polynomial equation and 3D plot results clearly demonstrate that the quadratic regression equation can demonstrate 3D response surface plots and estimate TPC, TFC, antioxidant activity (DPPH and FRAP assays), and carotenoid concentration in MFE samples (Figure 1A–O). Visually, the curvature of the response surfaces can be noticed, which reflects the degree of effect of the independent variables in the study values. Temperature (B), time (C), and ethanol concentration (A) showed a substantial effect on the biological properties and antioxidant activity of the MFE. Different shapes reflect various interactions between the factors being studied. If the contour plot was elliptical, the interactions between the corresponding variables would be very important; nevertheless, the circular contour plot suggests that there were no significant interactions between the variables [34]. From the RSM results, the optimum condition of ultrasound-assisted extraction for all antioxidants (total phenolic compound, total flavonoid content, total carotenoid content) and antioxidant activity (DPPH and FRAP assays) was a 68% (*v*/*v*) ethanol concentration, 40 °C temperature, and 15 min extraction time from the optimized RSM data. Based on the optimum results of the antioxidant constituents, antimicrobial activity by the disk diffusion method and minimum inhibitory concentration [30] on Gram-positive bacteria (*Staphylococcus aureus*) and Gram-negative bacteria (*Escherichia coli*) were further investigated.

### 3.2. Analysis of Bioactive Properties of MFE

**Figure 2:**
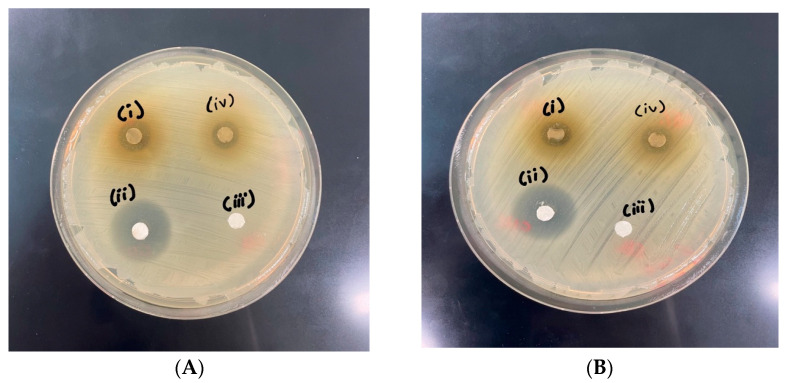
Antimicrobial activity analyzed by clear zone of pathogen inhibition, and minimum inhibitory concentration (MIC) of marigold flower extract (MFE) against (**A**) *Escherichia Coli*; (**B**) *Staphylococcus aureus*. The spots (i, ii, iii, iv) indicate 68-MFE: MFE extracted in 68% ethanol, 80-MFE: MFE extracted in 80% ethanol, chloramphenicol, and absolute ethanol inhibition zones, respectively.

MFE samples extracted with 68% and 80% ethanol (*v*/*v*), along with chloramphenicol and absolute ethanol, were analyzed for zone of inhibition and minimum inhibitory concentration (MIC) with *S. aureus* and *E. coli*. The diameter of the inhibition zones for the tested bacteria using the MFE samples against positive controls, such as chloramphenicol and absolute ethanol, are shown in Figure 2. For the crude extract, a maximal inhibition zone of 9.66 mm was measured in an *E. coli*-inoculated plate at the i-spot treated with a 68% extracted MFE sample (Figure 2A), whereas a minimum inhibition zone of 8.33 mm was measured in an *S. aureus* plate at the i-spot, as marked in Figure 2B. Following this, an iv-spot treated with an 80% extracted MFE sample inoculated with *E. coli* (Figure 2A) showed 6.8 mm; meanwhile, Figure 2B describes a plate with a 7.5 mm inhibition zone in an *S. aureus*-inoculated plate at the iv-spot. Additionally, the MFE sample extracted in 68% ethanol that showed the highest inhibition zones in the tested bacteria was analyzed. According to the results, the crude extract showed lower antimicrobial activities than the positive control, chloramphenicol, showed at ii-spots: 19.4 mm and 17.2 mm, respectively. It was found that the MIC values of *E. coli* and *S. aureus* were 0.25 mg/mL and 0.5 mg/mL, respectively. Consistently, the study of De Zoysa et al. [35] demonstrated that *S. aureus* (Gram-positive) has a greater antimicrobial capacity than *E. Coli* (Gram-negative). Gram-negative bacteria can cause resistance through alteration in the outer membrane, such as by modifying the hydrophobic character or causing mutations in porins [36].

### 3.3. Impact of Microencapsulation Process on Physicocpemical Properties and Microstructure of MFE Microcapsules

#### 3.3.1. Physicochemical Properties of GA- and MD-Based MFE Microcapsules

The percentage of yield obtained from the MFE microcapsules from spray drying was between 53.59 ± 0.70 and 79.2 ± 0.56 (*p* ≤ 0.05), as shown in Table 5. It was noted that the use of a 45% MD (1:2) sample gave the highest yield percentage (79.2%), compared to the GA samples (*p* ≤ 0.05). In addition, GA provided the lowest yield percentage (53.59%). Furthermore, the results indicated that the moisture content was in a range between 3.19 ± 2.05 to 4.35 ± 0.13, while the water activity was between 0.10 ± 0.04 and 0.15 ± 0.07. It was discovered that the water activity value was influenced by the growth of microorganisms that can cause food spoilage, specifically the growth of microbes which generate toxins that can harm consumers [19]. Pudziuvelyte et al. showed that increasing the concentration of maltodextrin and gum arabic led to a decrease in the moisture content of the microcapsules during spray drying, which is in agreement with our findings [37]. In this study, water activity for dried food products should be lower than 0.6 and moisture content should be less than 8%.

According to the color values of the MFE following spray drying, it was discovered that the coating material type and the encapsulation ratio affected the color values. The color values of the MFE microcapsules were explored after spray drying for L* (lightness), a* (green, red), and b* (blue, yellow). L* indicates brightness from 0 to 100, wherein 0 means black and 100 means white (very bright). All the samples had a negative a* value, representing that they were greenish. The MFE microcapsule using maltodextrin at a ratio of MFE and coating material 1:1 (*w*/*v*) had the highest a* value (−5.99). The MFE microcapsule using gum arabic as a coating material at a ratio of MFE and coating material 1:2 (*w*/*v*) had the highest yellowness value (30.45). A 45% MD (1:1) sample containing MFE displayed the highest brightness with 73.18 ± 1.51, in comparison with the 20% GA (1:2) sample which showed the least lightness (L* = 58.16 ± 3.81).

The encapsulation efficiency of the MFE microcapsules ranged from 78.05 ± 1.04 to 88.00 ± 1.15. It was found that the microcapsules using maltodextrin showed the highest encapsulation efficiency (88.00%), while those using gum arabic as an encapsulant resulted in (78.05%) efficiency. Our results are consistent with those of Murugesan and Orsat, who observed that encapsulating dry powder from elderberry juice (*Sambucus nigra L*.) with GA resulted in the lowest yield (59.26%), compared to using maltodextrin as the coating material in encapsulation [38]. The solubility of the MFE microcapsules ranged between 88.41 ± 2.91 and 92.98 ± 4.22%. Using maltodextrin as a coating material showed the highest encapsulation efficiency due to its properties in terms of good solubility and low viscosity at a high concentration. In addition, increasing the coating material ratio resulted in a higher encapsulation efficiency. It is possible that the thickness of the microcapsule could result in an improvement in the protection of the core material [13].

#### 3.3.2. Total Bioactive Analysis of GA- and MD-Based MFE Microcapsules

The TPC, TFC, TFC, and antioxidant activity according to the DPPH and FRAP assays of the MFE microcapsules obtained from different proportions of GA and MD are presented in Table 5. The amount of TPCs in 20% GAM (1:2), 20% GAM (1:3), 45% MDM (1:1), and 45% MDM (1:2) samples were 304.10 ± 7.06, 435.24 ± 8.32, 508.13 ± 9.52, and 627.91 ± 24.86 mg GAE/100 g db, respectively. The highest TPCs were obtained in the 20% GAM (1:2) sample, compared to the 45% MDM (1:2) sample, which showed the lowest values in all the microencapsulated samples. The total flavonoid content of all the microencapsulated samples was in the range of 209.87 ± 4.60 to 389.56 ± 9.58 mg QCE/100 g db. The 20% GAM (1:2) sample had the highest flavonoid content (389.56 ± 9.58 mg QCE/100 g db), while the 45% MDM (1:2) sample had the lowest amount of TFC (209.87 ± 4.60 mg QCE/100 g db). The TCC of all the microencapsulated samples was in the range of 44.62 ± 2.63 to 208.45 ± 2.36 mg carotenoid/100 g db. The highest TCC was attained in the 20% GAM (1:2) sample (208.45 ± 2.36 mg carotenoid/100 g db), in comparison with the 45% MDM (1:2) sample which showed the lowest TCC. Rajabi et al. found that using GA as a wall material and an encapsulating agent in the production of honey powder, amla extract, and basil leaves enhanced the phenolic content and antioxidant activity [39]. In the current study, a greater amount of total phenolic compound was found in samples with GA as the encapsulant. This is possibly due to the high polyphenol affinity of binding to GA; additionally, gum arabic is highly soluble with good emulsification and good film formation, which preserve polyphenols during processing [40].

The antioxidant activities of the MFE microcapsule samples measured by the DPPH and FRAP assays were in the range of 794.50 ± 15.61–1756.66 ± 28.99 mM Trolox equivalent/100 g db and 2665.96 ± 21.91–4837.89 ± 27.85 mM Trolox equivalent/100 g db, respectively. The highest DPPH and FRAP values were evidenced in the 20% GAM (1:2) sample, and the 45% MDM (1:2) sample had the lowest antioxidant values. The highest antioxidant activities according to the DPPH and FRAP assays were found in the sample that used GA as an encapsulant. Ferrari, Marconi Germer, Alvim, and de Aguirre have identified that applying gum arabic as an encapsulating agent provided higher antioxidant activity than maltodextrin in spray-dried blackberry juice, which is in agreement with our findings [30]. GA is able to promote the Maillard reaction, and its products are identified to improve antioxidant activity [41].

#### 3.3.3. Microstructural Analysis of GA- and MD-Based MFE Microcapsules

**Figure 3:**
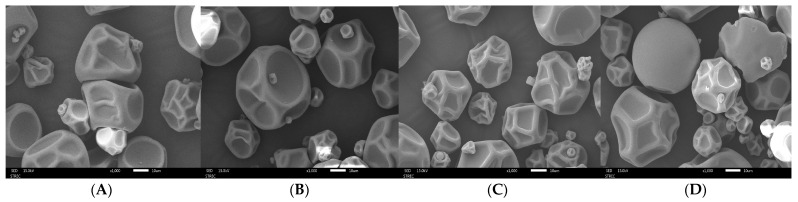
Scanning electron micrographs of marigold flower extract (MFE) microcapsules visualized at 1000× magnification. From left to right: (**A**) MFE encapsulated in 45% MDM (1:1, *w*/*v*); (**B**) MFE encapsulated in 45% MDM (1:2, *w*/*v*); (**C**) MFE encapsulated in 20% GAM: (1:2, *w*/*v*); (**D**) MFE encapsulated in 20% GAM (1:3, *w*/*v*). MDM: maltodextrin microcapsule; GAM: gum arabic microcapsule.

The scanning electron micrographs at a magnification of 1000× of GA and MD-based atomized microcapsules containing MFE are displayed in Figure 3. It was revealed that 20% GAM (1:3, *w*/*v*) samples had a spherical and dent-/crimp-free microcapsules shape (Figure 3D). The dents on the microcapsules were due to spray drying, which generated steam pressure on the interior structure, causing rapid shrinkage caused by moisture loss [16]. In contrast, the 20% GAM (1:2, *w*/*v*) sample containing a lower ratio of GA as the wall material produced microcapsules with surface constriction, as indicated in Figure 3C. When gum arabic was used as the coating material, it was discovered that the microcapsules with gum arabic at MFE and coating material ratios of 1:2 and 1:3 (*w*/*v*) were similar in shape; however, more dents and pleats were detected at the 1:2 (*w*/*v*).

MD microcapsules at varying ratios (1:1 and 1:2) of MFE to MD were different in terms of capsule appearance and size when compared to GA microcapsules. Increasing the MFE and MD ratio from 1:1 to 1:2 (*w*/*v*) resulted in smoother and more spherical microcapsules due to the increased encapsulation layer thickness. The contraction and deformation of spray-dried particulate are associated with temperature and liquid dispersion because longer drying times cause the configuration to change shape, shrink (resulting in roughness), and crumble (which results in breakage) [34]. The formation of concavities in the gum arabic powders may be a result of the particle shrinking that takes place due to rapid evaporation during the drying process [42]. In another study by Cano-Higuita et al. [43], microcapsules made entirely of gum arabic had smooth surfaces but some teeth on the surface that exhibited shrinkage, whereas microcapsules made from maltodextrin and modified starch had somewhat round surfaces with wrinkles on the surface but no fractures.

MFE encapsulated in 45% MD (1:1, *w*/*v*); MFE encapsulated in 45% MD (1:2, *w*/*v*); MFE encapsulated in 20% GA: (1:2, *w*/*v*); (MFE encapsulated in 20% GA (1:3, *w*/*v*). MDM; maltodextrin microcapsule, GAM; gum arabic microcapsule. Total phenolic compound (TPC), total flavonoid content (TFC), antioxidant activity by 2,2-diphenyl-1-picrylhydrazyl (DPPH) and ferric reducing antioxidant power (FRAP) assays, and carotenoid content.

## 4. Conclusions

MFE prepared via the RSM optimization of ethanol concentration (68%) (*v*/*v*), temperature (40 °C), and UAE time (15 min) revealed the highest bioactive compounds content and antioxidant activities. Among all the tested samples, the MFE sample extracted using 68% ethanol showed the highest zone of inhibition (9.66 mm and 8.33 mm) and MIC values (0.25 and 0.5 mg/mL) against *E. coli* and *S. aureus*, respectively. The MFE sample was encapsulated in 45% MD and 20% GA, and the highest yield (79.20%) was attained in the 45% MD (1:2) sample. Additionally, the moisture content, water activity, encapsulation efficiency, and solubility were higher in the 45% MD (1:2) sample, compared to other samples. However, the highest L*, a*, and b* values were visualized in 45% MD (1:1 ratio), 20% GA (1:3), and 20% GA (1:2), respectively. The MFE microcapsules in 20% GA (1:1) demonstrated the highest values of TPCs (627.91 mg GAE/100 g db), TFC (389.56 mg QCE/100 g db), TCC (208.45 mg carotenoid/100 g db), and antioxidant activity by the DPPH (1756.66 mM TE/100 g db) and FRAP (4837.89 mM TE/100 g db) assays, in comparison with other microcapsules. Moreover, the morphology of the MFE microcapsules visualized under SEM displayed fewer constrictions or dents with a bigger microcapsule size in 45% MD, and spherical-shaped microcapsules were seen in the 20% GA samples. In conclusion, the ratios of GA and MD played a vital role in the formulation of functional microcapsules containing MFE using spray drying technology. These bioactive microcapsules will be a value addition to the bio-circular economy and an essential ingredient for functional food development.

## Figures and Tables

**Table 1:** The values for the Box–Behnken design (BBD) using response surface methodology for the optimized extraction of marigold flower extract.

Independent Variables	Independent Variable Codes	Level		
		−1	0	1
Ethanol concentration (%)	A	60	80	100
Extraction temperature (°C)	B	30	40	50
Ultrasonication tim (min)	C	5	10	15

**Table 2:** The functional properties of marigold flower extract (MFE) under different extraction conditions using response surface methodology.

			Independent Variables			Responses		
Run	Concentration (%)	Temperature (°C)	Time (min)	TPC (mg GAE/ 100 g Dry wt.)	TFC (mg QE/ 100 g Dry wt.)	DPPH (mM Trolox/ 100 g Dry wt.)	FRAP (mM Trolox/ 100 g Dry wt.)	Carotenoid Content (mg Carotenoid/ 100 g Dry wt.)
1	80	50	15	72.38	95.73	610.50	4627.37	368.23
2	100	30	10	33.59	37.83	606.75	750.74	49.51
3	80	40	10	71.28	66.30	624.25	3364.21	288.62
4	80	40	10	70.29	47.68	625.50	2995.79	266.23
5	100	40	15	69.75	76.19	621.44	1722.11	137.96
6	80	40	10	74.14	49.36	643.62	2501.05	323.17
7	80	30	5	34.69	17.45	609.25	4585.26	414.67
8	80	40	10	72.71	57.83	644.87	2869.47	294.69
9	80	50	5	72.71	56.72	622.37	1869.47	312.94
10	100	50	10	74.14	55.73	627.37	7364.21	79.36
11	60	40	5	79.19	48.37	634.25	2880.00	292.48
12	100	40	5	91.94	92.14	623.31	1995.79	231.12
13	80	40	10	75.20	64.30	634.56	2932.63	266.23
14	60	50	10	69.31	55.24	615.81	9048.42	95.12
15	60	40	15	70.46	50.85	633.62	2427.37	55.04
16	60	30	10	31.39	14.78	581.12	1039.16	184.68
17	80	30	15	34.14	18.33	607.69	2422.32	319.30

Total phenolic compound (TPC), total flavonoid content (TFC), antioxidant activity by 2,2-diphenyl-1-picrylhydrazyl (DPPH), and ferric reducing antioxidant power (FRAP) assays and carotenoid content.

**Table 3:** Analysis of variance (ANOVA) for determination of optimization model fit.

	**TPC** **(mg GAE** **/100 g Dry wt.)**				**DPPH** **(mM Trolox** **/100 g Dry wt** **.)**			
**Source**	**Sum of Squares**	**df**	**Mean Square**	***p*** **-** **Value**	**Sum of Squares**	**df**	**Mean Squares**	***p*** **-** **Value**
Model	5361.08	9	595.68	0.0001	2879.70	9	319.97	0.16
A	45.44	1	45.44	0.17	24.72	1	24.72	0.69
B	2992.49	1	2992.49	0.0001	634.57	1	634.57	0.08
C	126.51	1	126.51	0.04	31.75	1	31.75	0.66
AB	1.74	1	1.74	0.77	49.44	1	49.44	0.58
AC	45.30	1	45.30	0.17	0.39	1	0.39	0.96
BC	0.01	1	0.01	0.98	26.59	1	26.59	0.69
A^2^	14.69	1	14.69	0.41	129.55	1	129.55	0.38
B^2^	2129.0	1	2129.0	0.0001	1901.32	1	1901.32	0.01
C^2^	44.24	1	44.24	0.17	3.11	1	3.11	0.89
Residual	132.75	7	18.96		1040.41	7	148.63	
Lack of Fit	116.64	3	38.88	0.03	663.45	3	221.15	0.21
Pure Error	16.11	4	4.03		376.95	4	94.24	
Cor Total	5493.84	16			3920.11	16		
R^2^				0.97				0.73
Adj-R^2^				0.94				0.39
	**FRAP** **(mM Trolox** **/100 g Dry wt** **.)**				**TFC** **(mg QE** **/100 g Dry wt** **.)**			
**Source**	**Sum of Squares**	**df**	**Mean Squares**	***p*** **-** **Value**	**Sum of Squares**	**df**	**Mean** **Squares**	***p*** **-** **Value**
Model	4.43 + 07	9	4.92 + 06	0.42	7147.90	9	794.21	0.04
A	1.59 + 06	1	1.59 + 06	0.56	1073.51	1	1073.51	0.05
B	2.49 + 07	1	2.49 + 07	0.04	3829.80	1	3829.80	0.0028
C	2157.21	1	2157.21	0.98	87.20	1	87.20	0.52
AB	4.87 + 05	1	4.87 + 05	0.74	127.21	1	127.21	0.44
AC	800.5 + 54	1	800.5 + 54	0.97	84.96	1	84.96	0.52
BC	6.05 + 06	1	6.05 + 06	0.27	363.47	1	363.47	0.21
A^2^	2.61 + 05	1	2.61 + 05	0.81	13.85	1	13.85	0.79
B^2^	7.89 + 06	1	7.89 + 06	0.21	1366.44	1	1366.44	0.03
C^2^	3.61 + 06	1	3.61 + 06	0.38	267.97	1	267.97	0.27
Residual	2.92 + 07	7	4.18 + 06		1326.53	7	1326.53	
Lack of Fit	2.89 + 07	3	9.62 + 06	0.0003	1040.66	3	346.89	0.08
Pure Error	3.80 + 05	4	95,124.65		285.86	4	71.47	
Cor Total	7.36 + 07	16			8474.43	16		
R^2^				0.60				0.84
Adj-R^2^				0.09				0.64
	**Carotenoid Content** **(mg Carotenoid** **/100 g Dry wt** **.)**							
**Source**	**Sum of Squares**		**df**		**Mean Squares**		***p*** **-** **Value**	
Model	1.89 + 05		9		20,967.33		0.01	
A	2091.99		1		2091.99		0.38	
B	1582.19		1		1582.19		0.44	
C	17,176.18		1		17,176.18		0.03	
AB	3565.06		1		3565.06		0.26	
AC	5205.23		1		5205.25		0.18	
BC	5674.03		1		5674.03		0.16	
A^2^	1.366 + 05		1		1.37 + 05		0.0001	
B^2^	127.09		1		127.09		0.82	
C^2^	21,519.70		1		21,519.70		0.02	
Residual	16,505.75		7		2357.96			
Lack of Fit	14,275.60		3		4758.53		0.03	
Pure Error	2230.15		4		557.54			
Cor Total	2.052 + 05		16					
R^2^							0.92	
Adj-R^2^							0.82	

Total phenolic compound (TPC), antioxidant activity by 2,2-diphenyl-1-picrylhydrazyl (DPPH). Total flavonoid content (TFC), antioxidant activity by ferric reducing antioxidant power (FRAP). Ethanol concentration (A), temperature (B), time (C). df: degree of freedom.

**Table 4:** The regression coefficient of the predicted second-order polynomial models (BBD) for bioactive properties.

	Bioactive Properties									
Factor	TPC	*p*-Values	TFC	*p*-Values	DPPH	*p*-Values	FRAP	*p*-Values	Carotenoid	*p*-Values
Intercept	72.73		57.10		634.56		2932.63		287.79	
Linear										
A	2.38	0.17	11.58	0.05	1.76	0.70	−445.26	0.56	−16.17	0.38
B	19.34	<0.0001	21.88	0.0028	8.91	0.08	1764	0.04	−14.06	0.44
C	−3.98	0.04	3.30	0.52	−1.99	0.66	−16.42	0.98	−46.34	0.03
Cross product										
AB	0.66	0.77	−5.64	0.44	−3.52	0.58	−348.95	0.74	29.85	0.26
AC	−3.37	0.17	−4.61	0.52	−0.31	0.96	44.74	0.97	36.07	0.18
BC	0.051	0.98	9.53	0.21	−2.58	0.69	1230.21	0.27	37.66	0.16
Quadratic										
A^2^	1.87	0.41	1.81	0.79	−5.55	0.38	249.10	0.81	−180.13	0.0001
B^2^	−22.49	<0.0001	−18.01	0.03	−21.25	0.009	1368.89	0.21	−5.49	0.82
C^2^	3.24	0.17	7.98	0.27	−0.86	0.89	−925.42	0.38	71.49	0.02

Total phenolic compound (TPC), total flavonoid content (TFC), antioxidant activity by 2,2-diphenyl-1-picrylhydrazyl (DPPH) and ferric reducing antioxidant power (FRAP) assays, and carotenoid content. Ethanol concentration (A), temperature (B), and time (C).

**Table 5:** Physicochemical and bioactive properties of gum arabic and maltodextrin microcapsules loaded with marigold flower.

	Treatments			
Parameters	20% GAM (1:2)	20% GAM (1:3)	45% MDM (1:1)	45% MDM (1:2)
Yield (%)	53.59 ± 0.70 ^d^	56.15 ± 0.73 ^c^	61.15 ± 0.58 ^b^	79.20 ± 0.56 ^a^
Moisture content (%)	4.42 ± 0.18 ^a^	3.45 ± 0.13 ^b^	4.35 ± 0.13 ^a^	3.19 ± 2.05 ^b^
Water activity	0.12 ± 0.03 ^ab^	0.15 ± 0.07 ^a^	0.12 ± 0.03 ^ab^	0.10 ± 0.04 ^b^
Color values				
L*	61.46 ± 1.55 ^c^	58.16 ± 3.81 ^d^	73.18 ± 1.51 ^a^	71.27 ± 1.39 ^b^
a*	−4.47 ± 0.17 ^b^	−3.17 ± 0.13 ^a^	−5.99 ± 0.00 ^c^	−5.23 ± 0.11 ^c^
b*	30.45 ± 1.59 ^a^	25.09 ± 1.53 ^b^	24.46 ± 0.61 ^c^	19.02 ± 0.73 ^d^
Encapsulation efficiency (%)	80.79 ± 1.12 ^b^	80.49 ± 1.85 ^b^	88.00 ± 1.15 ^a^	78.05 ± 1.04 ^c^
Solubility (%)	88.41 ± 2.91 ^bc^	92.73 ± 3.20 ^ab^	89.31 ± 1.31 ^b^	92.98 ± 4.22 ^a^
TPC (mg GAE/100 g dry wt.)	627.91 ± 24.86 ^a^	508.13 ± 9.52 ^b^	435.24 ± 8.32 ^c^	304.10 ± 7.06 ^d^
TFC (mg QE/100 g dry wt.)	389.56 ± 9.58 ^a^	376.73 ± 10.37 ^b^	282.90 ± 1.53 ^c^	209.87 ± 4.60 ^d^
Carotenoid content (mg carotenoid/100 g dry wt.)	208.45 ± 2.36 ^a^	162.75 ± 1.69 ^b^	55.22 ± 1.94 ^c^	44.62 ± 2.63 ^d^
DPPH (mM Trolox/100 g dry wt.)	1756.66 ± 28.99 ^a^	1469.70 ± 31.02 ^b^	1217.83 ± 22.68 ^c^	794.50 ± 15.61 ^d^
FRAP (mM Trolox/100 g dry wt.)	4837.89 ± 27.85 ^a^	4308.07 ± 26.49 ^b^	3076.49 ± 16.08 ^c^	2665.96 ± 21.91 ^d^

Three replications were used for each microcapsule per each analysis. Different letters (a, b, c, d) within same row indicate statistically significant differences (*p* ≤ 0.05).

## Data Availability

The original contributions presented in the study are included in the article, further inquiries can be directed to the corresponding author.
